# Female gender predicts lower access and adherence to antiretroviral therapy in a setting of free healthcare

**DOI:** 10.1186/1471-2334-11-86

**Published:** 2011-04-06

**Authors:** Christine Tapp, M-J Milloy, Thomas Kerr, Ruth Zhang, Silvia Guillemi, Robert S Hogg, Julio Montaner, Evan Wood

**Affiliations:** 1 British Columbia Centre for Excellence in HIV/AIDS, St. Paul's Hospital, Vancouver, Canada; 2 Department of Medicine, University of British Columbia, Vancouver, Canada

## Abstract

**Background:**

Barriers to HIV treatment among injection drug users (IDU) are a major public health concern. However, there remain few long-term studies investigating key demographic and behavioral factors - and gender differences in particular - that may pose barriers to antiretroviral therapy (ART), especially in settings with universal healthcare. We evaluated access and adherence to ART in a long-term cohort of HIV-positive IDU in a setting where medical care and antiretroviral therapy are provided free of charge through a universal healthcare system.

**Methods:**

We evaluated baseline antiretroviral use and subsequent adherence to ART among a Canadian cohort of HIV-positive IDU. We used generalized estimating equation logistic regression to evaluate factors associated with 95% adherence to antiretroviral therapy estimated based on prescription refill compliance.

**Results:**

Between May 1996 and April 2008, 545 IDU participants were followed for a median of 23.8 months (Inter-quartile range: 8.5 - 91.6), among whom 341 (63%) were male and 204 (37%) were female. Within the six-month period prior to the baseline interview, 133 (39%) men and 62 (30%) women were on ART (*p *= 0.042). After adjusting for clinical characteristics as well as drug use patterns measured longitudinally throughout follow-up, female gender was independently associated with a lower likelihood of being 95% adherent to ART (Odds Ratio [OR] = 0.70; 95% Confidence Interval: 0.53-0.93).

**Conclusions:**

Despite universal access to free HIV treatment and medical care, female IDU were less likely to access and adhere to antiretroviral therapy, a finding that was independent of drug use and clinical characteristics. These data suggest that interventions to improve access to HIV treatment among IDU must be tailored to address unique barriers to antiretroviral therapy faced by female IDU.

## Background

During the past decade, there have been significant advances in the treatment of HIV disease with the advent of antiretroviral therapy (ART) [1]. ART has been shown to suppress plasma HIV RNA, contributing to substantial reductions in HIV-related morbidity and mortality among people receiving treatment [2,3]. However, effective management of HIV disease requires high levels of ART adherence [4,5], as incomplete adherence can detrimentally affect virological control and subsequently disease progression, as well as contribute to elevated rates of antiretroviral resistance [5]. Therefore, ensuring that HIV-positive persons maintain high levels of ART adherence is of critical clinical importance.

Although newer, simplified ART regimens have enhanced treatment adherence [6], specific HIV-positive populations, such as injection drug users (IDU), continue to face barriers to accessing and adhering to ART. Recent injection drug use is associated with both non-adherence to ART and HIV disease progression [7,8], and many IDU live in unstable housing, have undiagnosed or untreated mental illness, high rates of incarceration, and street-involved survival-lifestyles, which may all complicate delivery of HIV-related treatments [9,10]. In addition, gender is an important and under studied variable that may explain barriers to effective HIV care. However, although previous research has assessed factors associated with access and adherence to ART among IDU, there remains a paucity of research investigating differences in adherence between male and female IDU specifically, as most of the previous research has explored adherence among either IDU generally or between non-IDU men and women [11-15]. There remain few long-term, prospective studies assessing gender as a factor that affects adherence conducted within a setting of universal healthcare.

British Columbia, Canada has a universally accessible, publicly funded healthcare system without user fees or other financial barriers to medical services, including all HIV/AIDS care. This allows for investigation of HIV-related outcomes without the potentially confounding effect of financial barriers to medical care and HIV treatment that may be present in other settings. Therefore, we conducted the present study to investigate factors associated with adherence to ART among a Canadian cohort of HIV-positive IDU and specifically examined if gender differences in adherence to ART existed in this context.

## Methods

The AIDS Care Cohort to evaluate Exposure to Survival Services (ACCESS) is a prospective observational cohort of HIV-seropositive injection drug users (IDU) in Vancouver, Canada. The cohort has been described in detail previously [14,16,17], and was populated through snowball sampling and extensive street outreach methods in the city's Downtown Eastside. Individuals were eligible for ACCESS if they were aged 18 years or older, HIV seropositive, had used injection drugs, and provided written informed consent. At baseline and semi-annually, participants answer a standardized interviewer-administered questionnaire and provide blood samples for serologic analysis.

As previously described [14,16,17], the local setting is somewhat unique in that there is a universal healthcare system and a province-wide centralized antiretroviral dispensation program and HIV/AIDS laboratory which enables a complete prospective profile of all patient CD4 cell count determinations and plasma HIV-1 RNA levels, as well as a complete prospective profile of antiretroviral therapy use among cohort participants. This includes the specific antiretroviral agents and the prescribed dose, a validated measure of patient adherence derived from prescription refill compliance, which is described further below [17,18]. The study has been approved by the Providence Health Care/University of British Columbia Research Ethics Board. Plasma HIV-1 RNA was measured using the Roche Amplicor Monitor assay (Roche Molecular Systems, Mississauga, Canada).

In the present study, we included all participants who were recruited and completed at least one interview between May 1996 and April 2008, and excluded only those where clinical data were unavailable. As indicated above, the primary independent variable of interest was gender and, to compare rates of ART use at baseline, we evaluated access to ART at the time of recruitment into the cohort by examining the proportion of participants who had been on ART in the 6-month period prior to the baseline interview. Since we had long-term prospective data, we then assessed the longitudinal pattern of ART exposure by examining ART adherence during the six-month period preceding each semi-annual follow-up visit throughout follow-up. As in previous studies using this validated and confidential pharmacy dispensation data [17,18], we measured adherence to therapy in each six-month period as a ratio of the number of days ART was dispensed over the number of days an individual was eligible for ART and defined adherence as equal to or greater than 95% adherence to ART during this period.

We considered explanatory variables potentially associated with the dependent variable including: gender (female vs. male); age (<24 yrs vs. ≥24 yrs); ethnicity (Aboriginal ancestry vs. other); daily injection heroin use (yes vs. no); daily cocaine injection (yes vs. no); daily crack cocaine smoking (yes vs. no); current methadone use (yes vs. no); any treatment use (yes vs. no); education (less than high school vs. other); employment (reg job, temp work, self-emp vs. other); and unstable housing (yes vs. no). Age was defined as a dichotomous variable according to the World Health Organization's definition of a 'young person', using the upper age limit of 24 as the cut-off [19]. All dichotomous behavioural variables referred to the six-month period prior to the interview. As in our previous work [18], we defined unstable housing as living in a single-room occupancy hotel, shelter or being homeless. Clinical variables included baseline HIV-1 RNA level (per log_10_copies/mL) and CD4 cell count (per 100 cells/mm^3^), using the closest measure within one year of the baseline.

We began by comparing the rate of ART use at baseline. We then examined univariate associations between the explanatory variables and ART adherence throughout follow-up. Because serial measures for each variable were available for each subject, we used generalised estimating equations (GEE) for the analysis of correlated data. This approach allows for the identification of factors associated with the outcome over the entire study period. Data from every participant follow-up visit were considered in this analysis. Missing data were addressed through the GEE estimating mechanism which uses all available pairs method to encompass the missing data from dropouts or intermittent missing. All non-missing pairs of data are used in the estimators of the working correlation parameters. Standard errors were calculated using an exchangeable correlation structure, adjusted by multiple observations for each individual. GEE models have routinely been used to analyse datasets containing repeated measures, including longitudinal IDU cohorts [20,21].

Following examination of the univariate results we fit a multivariate GEE logistic regression model using an *a priori *defined model building protocol whereby we included all explanatory variables with a univariate *p*-value < 0.05. We also ran the models with interaction terms for key independent variables in order to better compare rates of adherence between men and women. We conducted additional sub-analyses adjusting for key clinical or demographic characteristics and for the year of the baseline interview to account for advances in antiretroviral therapy during the study period. All statistical procedures were performed using SAS version 9.1 (SAS, Cary, NC, USA). All *p*-values are two-side.

## Results

Between May 1996 and April 2008, 545 participants were eligible for the present study, and there was a median of 23.8 months (Inter-quartile range: 8.5 - 91.6) of prospective follow-up. Of these study participants, 341 (63%) were male and 204 (37%) were female. The characteristics of the study population stratified by gender are shown in Table 1. As indicated in the table, 133 (39%) men and 62 (30%) women were on ART in the six-month period prior to the baseline interview (*p *= 0.042). There were 1186 (26.6%) periods where individuals were adherent out of 4460 total observations. Overall, 81 (19%) out of 422 males and 83 (29%) out of 287 females were excluded from the analysis as a result of missing baseline CD4 count or viral load.

**Table 1:** Baseline socio-demographic, behavioural, and clinical characteristics of ACCESS participants^†^, stratified by gender.

Characteristic	Male *n = 341 (63%)*	Female *n = 204 (37%)*	Odds Ratio (95% CI)	*p *value
**Age**				
≥ 24 years	332 (97)	179 (88)	5.2 (2.4-11.3)	<0.001
<24 years	9 (3)	25 (12)		
**Aboriginal ethnicity**				
No	249 (73)	111 (54)		
Yes	92 (27)	93 (46)	2.3 (1.6-3.3)	<0.001
Daily heroin use*				
No	264 (77)	129 (63)		
Yes	77 (23)	75 (37)	2.0 (1.4-2.9)	<0.001
Daily cocaine use*				
No	227 (67)	126 (62)		
Yes	114 (33)	78 (38)	1.2 (0.9-1.8)	0.256
Daily crack use*				
No	271 (79)	142 (70)	1.7 (1.1-2.5)	0.009
Yes	70 (21)	62 (30)		
**Current methadone use**				
No	251 (74)	125 (61)		
Yes	90 (26)	79 (39)	1.8 (1.2-2.6)	0.003
**Any treatment**				
No	140 (41)	75 (37)		
Yes	201 (59)	129 (63)	1.2 (0.8-1.7)	0.320
**Unstable housing**				
No	95 (28)	64 (31)		
Yes	246 (72)	140 (69)	0.8 (0.6-1.2)	0.383
**On ART at baseline**				
No	208 (61)	142 (70)		
Yes	133 (39)	62 (30)	0.7 (0.5-1.0)	0.042
**Viral load (log**_**10**_**copies/mL)**				
≥ 100,000	66 (19)	32 (16)	1.3 (0.8-2.1)	0.281
<100,000	275 (81)	172 (84)		
**CD4+ count (cells/mm**^**3**^**)**				
≥ 200	261 (77)	164 (80)	0.8 (0.5-1.2)	0.294
<200	80 (23)	40 (20)		

^†^Includes all eligible participants that completed at least one interview between May 1996 and April 2008 and where baseline clinical data was available. *Refers to the six-month period prior to the baseline interview.

The results of the univariate GEE logistic regression demonstrated that methadone use (Odds Ratio [OR] = 2.44 [95% CI: 2.01-2.96]; *p *< 0.001), and accessing any addiction treatment (OR = 1.50 [95% CI: 1.26-1.79]; *p *< 0.001) were associated with being 95% adherent to ART, whereas higher baseline viral load (OR = 0.54 [95% CI: 0.48-0.60]; *p *< 0.001), education (OR = 0.56 [95% CI: 0.37-0.85; *p *= 0.007] age less than 24 years (OR = 0.16 [95% CI: 0.08-0.31]; *p *< 0.001), daily heroin injection (OR = 0.38 [95% CI: 0.30-0.48]; *p *< 0.001), daily cocaine injection (OR = 0.48 [95% CI: 0.40-0.57]; *p *< 0.001), and female gender (OR = 0.69 [95% CI: 0.52-0.90]; *p *= 0.006) were negatively associated with 95% adherence. Table 2 outlines the results of the univariate GEE analysis.

**Table 2:** Univariate GEE*^ϕ ^analysis of sociodemographic, behavioural and clinical factors associated with ≥ 95% ART adherence.

Variable	OR	(95% CI)	*p*-value
**Gender**			
(Female vs. Male)	0.69	(0.52-0.90)	0.006
**Age**			
(<24 yrs vs. ≥ 24 yrs)	0.16	(0.08-0.31)	<0.001
**Ethnicity**			
(Aboriginal vs. other)	0.97	(0.74-1.28)	0.841
Heroin use*			
(Daily vs. not daily)	0.38	(0.30-0.48)	<0.001
Cocaine use*			
(Daily vs. not daily)	0.48	(0.40-0.57)	<0.001
Crack use*			
(Daily vs. not daily)	0.94	(0.78-1.13)	0.509
**Methadone treatment**			
(Yes vs. no)	2.44	(2.01-2.96)	<0.001
**Other addiction treatment**^**†**^			
(Yes vs. no)	1.50	(1.26-1.79)	<0.001
**Education**			
(less than highschool vs. other)	0.56	(0.37-0.85)	0.007
**Employment**			
(reg job, temp work, self-emp vs. other)	1.11	(0.87-1.41)	0.401
**Unstable housing**			
(Yes vs. no)	0.90	(0.76-1.07)	0.239
**Viral load**			
(per log_10_copies/mL)	0.54	(0.48-0.60)	<0.001
**CD4+ count**			
(per 100 cells/mm^3^)	0.95	(0.89-1.00)	0.047

*Refers to the previous six-month period. ^†^Defined as drug treatment other than methadone in the past six months. ^ϕ^GEE = Generalized Estimating Equation.

**Figure 1:**
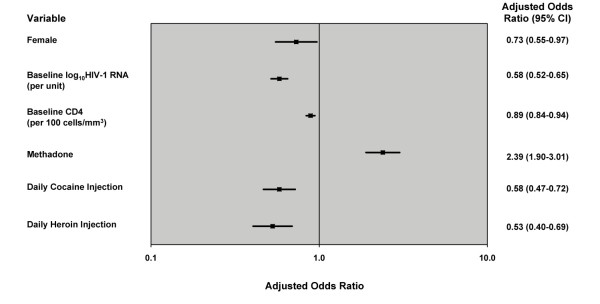
Factors independently associated with 95% ART adherence among HIV-positive injection drug users in GEE* logistic regression. *GEE = Generalized Estimating Equation. Multivariate model fit.

Multivariate GEE regression demonstrated that methadone use (OR = 2.35 [95% CI: 1.88-2.94]; *p *< 0.001) was independently associated with 95% ART adherence. Higher baseline viral load (OR = 0.81 [95% CI: 0.68-0.97]; *p *= 0.018), age less than 24 years (OR = 0.27 [95% CI: 0.13-0.57]; *p *< 0.001), daily heroin injection (OR = 0.56 [95% CI: 0.43-0.73]; *p *< 0.001), daily cocaine injection (OR = 0.57 [95% CI: 0.47-0.71]; *p *< 0.001); education (OR = 0.65 [95% CI: 0.43-0.98]; *p *= 0.04); and, female gender (OR = 0.70 [95% CI: 0.53-0.93]; *p *= 0.013) were independently and negatively associated with 95% adherence to ART. There were no statistical interactions observed in the multivariate GEE analysis. Table 3 and Figure 1 show results of the multivariate GEE logistic regression.

**Table 3:** Multivariate GEE*^ϕ ^analysis of sociodemographic, behavioural and clinical factors associated with ≥ 95% ART adherence.

Variable	OR	(95% CI)	*p*-value
**Gender**			
(Female vs. Male)	0.70	(0.53-0.93)	0.013
**Age**			
(<24 yrs vs. ≥ 24 yrs)	0.27	(0.13-0.57)	<0.001
Heroin use*			
(Daily vs. not daily)	0.56	(0.43-0.73)	<0.001
Cocaine use*			
(Daily vs. not daily)	0.57	(0.47-0.71)	<0.001
**Methadone treatment**			
(Yes vs. no)	2.35	(1.88-2.94)	<0.001
**Education**			
(less than highschool vs. other)	0.65	(0.43-0.98)	0.04
**Viral load**			
(per log_10_copies/mL)	0.81	(0.68-0.97)	0.018
**CD4+ count**			
(per 100 cells/mm^3^)	0.89	(0.84-0.94)	<0.001

*Refers to the previous six-month period. ^†^Defined as drug treatment other than methadone in the past six months. ^ϕ^GEE = Generalized Estimating Equation.

We conducted several sub-analyses of behavioural and clinical variables. A sub-analysis was conducted defining drug use as a 6-level categorical variable using 'daily injecting' as the reference level showed results that were consistent with analyses defining drug use as a dichotomous variable (data available from the corresponding author). Sub-analyses adjusting for year of the baseline interview, to account for advances in antiretroviral therapy during the study period, were consistent with the primary analysis (data available from the corresponding author). Additionally, a sub-analysis of time-updated clinical variables to determine if clinical outcomes affected adherence or drug using behaviours demonstrated that, although cocaine use was no longer significant, the gender effect remained (OR = 0.56 [95% CI: 0.43-0.74]; *p *< 0.001).

## Discussion

The present study demonstrates that female IDU are approximately 30% less likely to adhere to ART, an association that persisted after intensive covariate adjustment. To our knowledge this is the first long-term study to assess key demographic and behavioural factors associated with ART adherence within a community-recruited cohort and within a context of a universal healthcare system, and implies that barriers to adherence among female IDU that are not explained by financial barriers.

Although female gender remained significantly associated with worse adherence in multivariate analyses, it is noteworthy that the strength of the association diminished when we adjusted for drug using and other behavioural variables. This implies that drug-using characteristics, which may create barriers to ART adherence, were more common among female IDU in our setting and this was confirmed by our baseline gender comparisons. These data suggest that women are more engaged in street-involved, survival activities, and are thereby more marginalized from the healthcare system, which poses a barrier to ART access and adherence.

The importance of maintaining high levels of ART adherence is well established [4,5], and ensuring that vulnerable and disadvantaged populations have equal access to HIV treatment is crucial from both an individual and public health standpoint [3,22]. For example, recent research from the COHERE cohort in Europe indicates that IDU and women, and particularly female IDU, experience higher mortality rates even after achieving optimal CD4 cell counts while on ART, underscoring the urgent health needs of this population and the importance of maintaining high rates of adherence [23]. However, there are currently few long term studies pertaining to factors associated with ART adherence among IDU, particularly in settings where HIV care is provided free of charge. Previous research regarding ART adherence across multiple populations in the U.S.A. found that, in a post-ART era, socio-demographic and behavioural factors - such as lower education status, lower income, lack of medical care coverage, a history of frequent drug or alcohol use, African-American race, and female gender - are more frequently associated with access and adherence to ART than clinical factors [12,24,25]. Importantly, female gender has been shown to be associated with lower rates of ART adherence across several sub-groups, including both IDU and non-IDU [15,25,26]. Compounding this, a national U.S. study found that female IDU were among the most disadvantaged sub-groups of vulnerable HIV-positive populations and were about half as likely to receive ART compared to homosexual males, even after adjusting for other socio-demographic variables [27]. However, because these studies were conducted in the U.S., financial and other barriers inherent to the American healthcare system make it difficult to fully determine whether other factors may be affecting the association with gender and other socio-demographic characteristics. The present study enhances the current body of research by demonstrating that gendered barriers to ART adherence persist even in settings with a universal healthcare system.

Research regarding methods to reduce barriers to ART are limited, particularly concerning gendered barriers, though the need for targeted interventions is clear [23]. Some studies have suggested that health system changes such as educating health care providers about the disparities that exist among populations accessing treatment, anonymous HIV testing and treatment sites, and same-day clinic appointments or extended hours of operation, may increase access and adherence to ART among vulnerable, marginalized populations [24,27]. In addition, providing women-only health and community-based services, increasing efforts to improve self-efficacy among women in engaging with the healthcare system, as well as education dispelling misinformation and misconception about HIV-treatment, may all contribute to improving access and adherence among women and female IDU, specifically [24,28]. However, as our analyses demonstrate, drug using characteristics and the corollary of more survival-based activities, are also a significant barrier to ART adherence among women and are likely a first point for interventions that seek to improve access to healthcare services among IDU women.

There are some limitations to this study. Most importantly, as this is an observational study, the association between gender and ART adherence should be interpreted with caution. It is possible that there are other confounding factors that were not measured and adjusted for in this study. However, our analysis included a large number of explanatory variables and we used a liberal *a priori *defined model fitting protocol [18]. In addition, our measure of adherence was based on prescription refill compliance, which measures something different than daily pill taking. With respect to this concern, we note that our measure of ART adherence has been shown to predict virological suppression [29], CD4 response [30], and mortality [17,18] and thus the differences we observed are likely to be clinically significant. Lastly, we do not feel that missing data played a role in our results given that all non-missing pairs of data are included as a result of using generalized estimating equation logistic regression in the statistical analysis when employing generalized estimating equation (GEE) logistic regression.

## Conclusions

The current study demonstrates that female gender presents an additional barrier for access and adherence to ART among injection drug users, independent of drug use and other socio-behavioural and clinical characteristics. This is evidence that even within a context where medical care and ART are provided free to HIV-positive individuals, there remain important healthcare access differences between male and female IDU. Other socio-structural factors are also critically important in affecting equitable access to necessary HIV treatment [31], and women often face barriers derived from their comparatively lower socio-economic status, and broader, systemic inequities that persist even within a context of universal healthcare. In order to improve access and adherence to ART among IDU women, gender-specific interventions should be developed which recognize these unique barriers.

## Competing interests

Conflict of Interest Disclosure Statement for J Montaner

Julio Montaner is supported by the Ministry of Health Services and the Ministry of Healthy Living and Sport, from the Province of British Columbia; through a Knowledge Translation Award from the Canadian Institutes of Health Research (CIHR); and through an Avant-Garde Award (No. 1DP1DA026182-01) from the National Institute of Drug Abuse, at the US National Institutes of Health. He also received funding from Merck, Gilead and ViiV to support research into Treatment as Prevention.

## Authors' contributions

EW, CT, and MJM drafted the manuscript. RZ performed the statistical analysis.

All authors read and approved the final manuscript.

## Pre-publication history

The pre-publication history for this paper can be accessed here:

http://www.biomedcentral.com/1471-2334/11/86/prepub
